# Model development and update of portable NIRS instrument for assessment of internal quality attributes of two navel orange varieties

**DOI:** 10.3389/fnut.2022.976178

**Published:** 2022-08-24

**Authors:** Xudong Sun, Di Deng, Jiacheng Liu, Shaoran Feng

**Affiliations:** ^1^School of Mechatronics and Vehicle Engineering, East China Jiaotong University, Nanchang, China; ^2^Ministry of Education, Key Laboratory of Conveyance Equipment (East China Jiaotong University), Nanchang, China; ^3^Cultivation Laboratory, Ganzhou Citrus Research Institute, Ganzhou, China; ^4^Beijing Sunlight Yishida Technology Co., Ltd., Beijing, China

**Keywords:** navel orange, SSC, TA, near infrared spectroscopy, batch, portable instrument

## Abstract

The variation of fruit among batches influences the performance of the portable near infrared spectroscopy (NIRS) instrument and then determines the success or failure for practical application in fruit industry. Model development and update methods were investigated for determining soluble solids contents (SSC) and titrable acidity (TA) of navel orange. The pretreatment and variable selection methods were explored for building partial least square regression (PLSR) models. The best models, developed by the combination of second derivative (2D) and variable sorting for normalization (VSN), could predict SSC but not TA. The root mean square error of prediction (RMSEP), coefficient of determination for prediction ($R_{\text{p}}^{2}$) and ratio of prediction to deviation (RPD) for SSC were 0.66 °Brix, 0.66 and 1.73. Model maintain methods of model update (MU) and slope and bias correction (SBC) achieved the best results in predicting SSC for two external validation sets with $R_{\text{p}}^{2}$, RMSEP and RPD of 0.54, 0.83 °Brix, 1.60 and 0.52, 0.83 °Brix, 1.65, respectively. The results suggested model development and update with MU and SBC could improve the robustness of the portable NIRS instrument in predicting SSC of navel orange.

## Introduction

The internal quality attributes of soluble solids content (SSC) and titrable acidity (TA) are main indexes for characterizing maturity level and taste of citrus fruit. The near infrared spectroscopy (NIRS) with the advantages of non-destructive, rapid and on field application was firstly integrated into package system for fruit sorting in Japan since the late 1980 s (1). The portable NIRS instrument followed online sorting system was developed successfully in 2,000, which was suitable for determining fruit properties such as monitoring quality variation for supply chain or predicting optimal harvest date (OHD) (2). To some extent, model development and update determine the success or failure for practical application of NIRS technology.

Currently, partial least square regression (PLSR) algorithm is adopted widely to build a robust model combined with pretreatment methods (3). The first or second derivative spectra were frequently applied to develop PLSR models rather than raw absorption spectra because of baseline correction with derivative pretreatment. The combinations of derivative spectra and standard normal variate (SNV) or multiplicative scattering correction (MSC) were generally operated for removing light scatter and improving the accuracy of the model to the interest attributes of fruit (4). An extension algorithm of SNV named variable sorting for normalization (VSN) was proposed by Rabatel et al. (5) in 2019. VSN adjusted the weight to the wavelength for strengthening the target variables. The root mean square error of prediction (RMSEP) was reduced by 34% with VSN pretreatment for olive dry matter content (DMC) when the size of fruit did not match with the measurement window of the portable NIRS instrument (6). The normalized spectral ratio (NSR) was proposed by Li et al. (7) in 2020, which chose two wavelengths for replacing slope and offset parameters of MSC. The RMSEP of SSC for apple was decreased from 0.85 to 0.64% after NSR correction using an online sorting system.

Variable selection also is a critical step for extracting useful variables or eliminating variables containing mostly noise during model development (3). Recently, variable selection algorithms were explored such as variable combination population analysis (VCPA) (8), iteratively retaining informative variables (IRIV) (9), Monte Carlo uniformation variable elimination (MC-UVE) (10), successive projections algorithm (SPA) (11) and window search (6). For fruit quality assessment by the portable NIRS instrument, an optimal wavelength region often was recommended. For example, a good result with determination coefficient (*R*^2^) of 0.90–0.96 and RMSEP of 0.29–0.33 °Brix was achieved in predicting SSC of pear, and the range 700–930 nm was recommended for pear sugar content using Vis-NIR instrumentation adopted in the current study (12). The region of 729–975 nm was recommended to build PLSR model for DMC of mango for Vis-NIR instrumentation (13). Therefore, variable selection methods should be considered in this work.

Mathematical models imbedded in the portable NIRS instrument performed poorly when the calibration model predicted new groups with different season, variety, population or environmental conditions (14). At present, the common solution is merging new samples into the original calibration set and recalibrated, here it is named as model updating (MU) (15). MU is necessary to support the portable NIRS instrument application success for fruit quality assessment (16). The variation between calibration and prediction sets causes the change of both slope and bias of PLSR model (17), slope and bias correction (SBC) is a simple and easy way to maintain PLSR model (18). The dynamic orthogonalization projection (DOP) algorithm adopts external parameter orthogonalisation (EPO) framework to correct the spectra between the original calibration and new prediction sets for updating the PLSR model. RMSEP was reduced by 66% in predicting DMC of olive fruit using DOP algorithm (19). The orthogonal signal correction (OSC) proposed by Wold et al. (20) provides another way to correct the spectra difference among fruit varieties (21). Mishra and Woltering (15) proposed a semi-supervised parameter-free calibration enhancement (PFCE) approach to update the model for predicting several new batches of pear and kiwi fruit. The performance of the model in predicting moisture content and total soluble solids of pear and kiwi fruit were greatly improved using PFCE approach. Therefore, Model updating also was considered for assessment the quality of navel orange.

The objective was to investigate an approach for model development and update in assessment the quality of nave orange using a portable NIRS instrument. The pretreatment and variable selection methods were attempted to build a robust PLSR model. The update methods were explored for improving the performance of PLSR model to new populations.

## Materials and methods

### Samples preparation

The populations of 1-6 (Newhall navel orange) were harvested from a local orchard from Oct. 14, 2021 to Dec. 31, 2021 located at Ganzhou Citrus Research Institute (114°51′2″E, 25°46′36″N), Ganzhou, China. The fruit tree was 6-year-old Newhall navel orange, a mainstream species of citrus. Thirty and forty samples were collected randomly from three trees at moderate height for the first five and sixth batches, respectively. The samples were cleaned and transported to the laboratory at Nanchang, China. Removing damage samples, a total of 179 navel orange samples were obtained. To verify the applicability of the model to new batches, the populations of 7–12 (Lunwan navel orange) (*n* = 180) were collected randomly from a local market from May 10, 2022 to Jul. 10, 2022. The statistical results for the samples were listed in Table 1. For understanding the differences and similarities well-between the two varieties, the main features for Newhall and Lunwan were listed in Table 2.

**Table 1:** Statistical results for 12 populations.

**Population**	**Variety**	**Fruit #**	**Spectra #**	**SSC (** **°** **Brix)**		**TA (g/L)**		**Date**
				**Mean**	**SD**	**Mean**	**SD**	
1	Newhall	22	66	11.3	0.77	101.1	22.64	14/10/2021
2	Newhall	29	87	11.2	0.92	88.8	14.56	24/10/2021
3	Newhall	29	87	12.1	1.02	18.4	6.00	09/11/2021
4	Newhall	29	87	11.9	1.07	17.4	6.65	23/11/2021
5	Newhall	30	87	12.6	1.14	17.8	7.21	10/12/2021
6	Newhall	40	120	12.5	0.87	16.0	4.38	31/12/2021
7	Lunwan	30	90	11.7	1.84			10/05/2022
8	Lunwan	30	90	11.8	1.27			20/05/2022
9	Lunwan	30	90	11.0	1.03			28/05/2022
10	Lunwan	30	90	11.3	1.22			10/06/2022
11	Lunwan	30	90	11.1	1.15			24/06/2022
12	Lunwan	30	90	11.2	1.10			10/07/2022
Total	359	1074					

*SSC is soluble solids content; TA is titrable acidity; SD is standard deviation*.

**Table 2:** The differences and similarities between two varieties derived from www.baidu.com with keywords of Newhall and Lunwan in Chinese.

**Variety**	**Weight**	**Mature period**	**Edible rate**	**Skin color**
Newhall	250–350 g	November to January	73–75%	Orange yellow
Lunwan	>200 g	March to April	74.1%	Shallow orange

*Weight refers to average weight of single fruit*.

**Figure 1:**
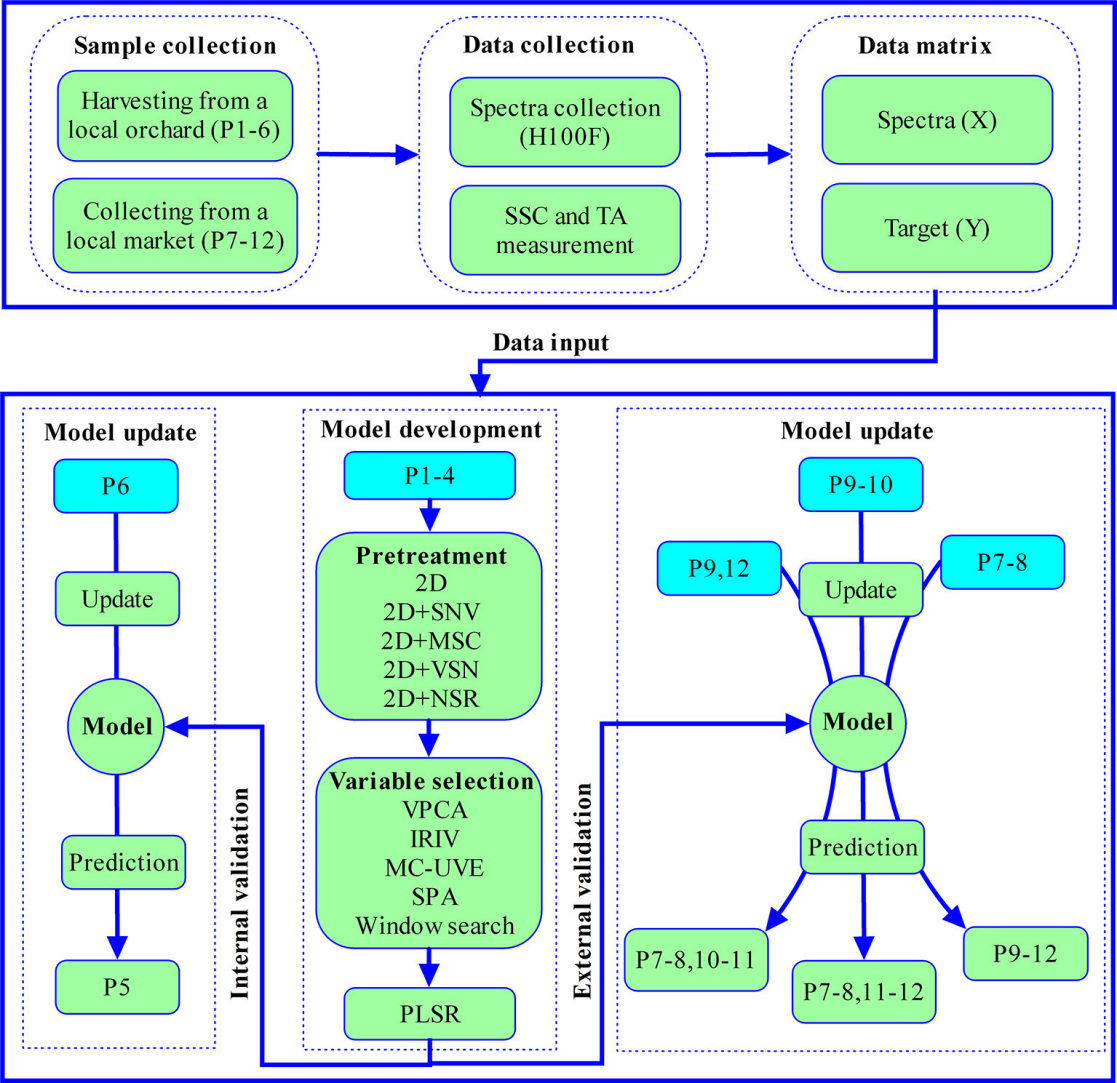
Flowchart of all experimental procedures, P represented population.

The procedures of the model development and update were shown in Figure 1. One internal and three external validation sets were applied to investigate influence of coverage of the update set to the prediction set (Figure 1).

### Near infrared spectra collection

**Figure 2:**
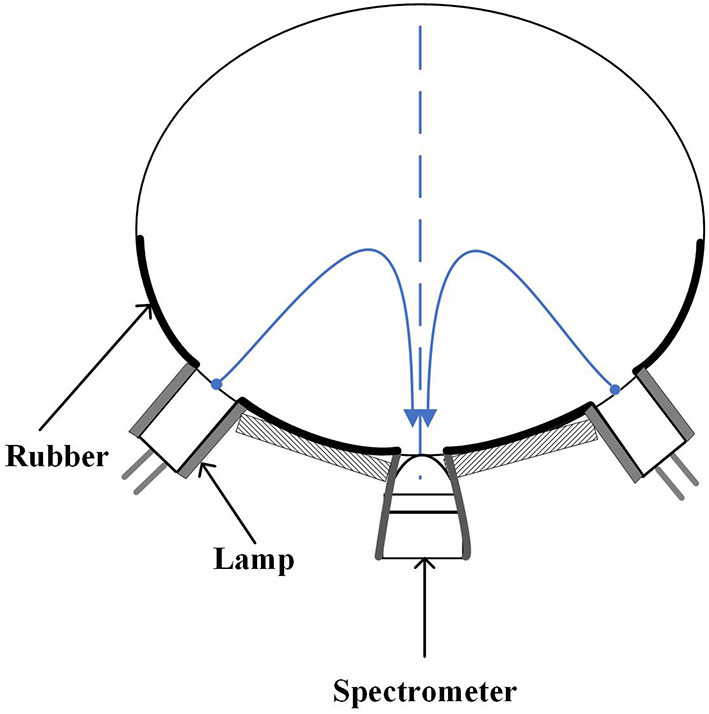
Optical geometry of H100F instrument.

The spectra of the samples were collected by hand-held non-destructive detector for fruit internal quality (H100F, Sunforest, Incheon, Korea). The full region from 650 to 950 nm composed of 151 variables with wavelength interval of 2 nm. The light source is two tungsten halogen lamps (10 W) arranged at an included angle of 100 degrees. In order to avoid the influence of external environment, the samples were placed at room temperature (about 20°C) for 24 h before spectra collection. After startup of the instrument, the reference and dark current spectrum were recorded with the integration time of 900 milliseconds. Then the spectrum of navel orange was recorded with the integration time of 900 milliseconds. During collection, the navel orange was tightly attached to the probe. The light emitted by two halogen tungsten lamps, after diffuse reflected by navel orange tissue, entered into the detector of the spectrometer and was converted into absorption spectrum (Figure 2). A spectrum was recorded every 120 degrees along the equator. Considering the variation of SSC distribution within a sample, each spectrum was regarded as a sample.

### Internal quality attributes measurement

After spectra collection, the flesh at the spectral measurement point was juiced, filtered and dripped into a beaker for measurement of SSC and TA. The SSC was measured by digital sugar meter (PAL-1, Atago Co., Tokyo, Japan). The measurement process is to suck juice drops onto the light window of the sugar meter until it covered the light window. The measurement was repeated for three times and the average value was obtained. TA was measured by potentiometric titration (PE28, Mettler-Toledo, Zurich, Switzerland), the end point of titration was reached when sodium hydroxide solution was used to titrate to pH 8.2, and TA was calculated according to the volume of sodium hydroxide solution consumed [formula (1)]. But it should be noted, for populations of 7–12 only SSC was measured.


(1) $$\begin{array}{r} X=\frac{\left[c\times \left(V_{1}-V_{2}\right)\right]\times k\times F}{m}\times 1000 \end{array}$$

Where *X* is *TA* value, *c* is the concentration of sodium hydroxide solution, *V*_1_ is the volume of sodium hydroxide solution consumed when titrating fruit juice, *V*_2_ is the volume of sodium hydroxide solution consumed during blank experiment, *k* is the conversion factor of acid (citric acid is 0.064), *F* is the dilution ratio of fruit juice and *m* is volume of juice.

### Data analysis

The pretreatment algorithms of second derivative (2D), SNV, MSC, VSN and NSR, variable selection algorithms of VCPA, IRIV, MC-UVE, SPA and window search and model upgrade algorithms of SBC, DOP and OSC were carried out by use of Matlab 2020a (Mathworks Inc., Natick, MA). The VSN and DOP algorithms were provided by Prof. Roger (5, 19), the others were programmed by ourselves. All the algorithms were programmed based on PLSR.

The 5-fold cross validation was adopted to determine the number of latent variables (LVs). The determination coefficient of cross validation and prediction (R${}_{\text{cv}}^{2}$ and R${}_{\text{p}}^{2}$), root mean square error of cross validation and prediction (RMSECV and RMSEP) were calculated. A quick guideline of RPD defined as the ratio of standard deviation (SD) to the RMSECV or the RMSEP was applied for model assessment (22, 23). The critical limit of RPD is one, above which some predictive power exists. Ideally, RPD should be >2 for a good calibration.

## Results and discussion

### Analysis of near infrared spectral characteristic

**Figure 3:**
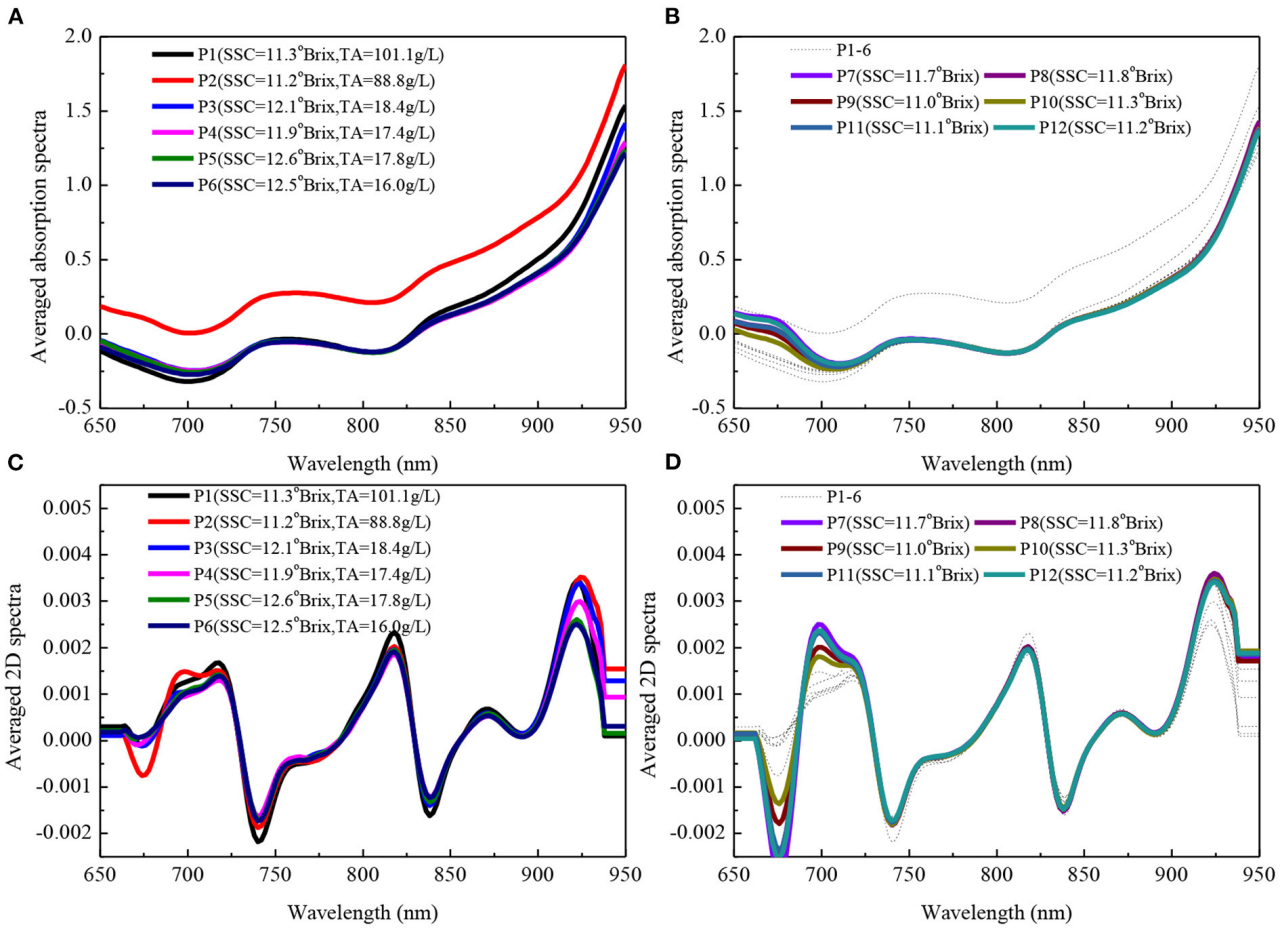
Averaged absorption **(A,B)** and 2D **(C,D)** spectra for 12 populations. For better comparison with P7–12, P1–6 was represented with a thin, black and dashed line in panel **(B,D)**. P represented population.

Generally, the derivative spectra were applied to build models for removing baseline shift in fruit quality assessment by the portable NIRS instrument (3). The spectra of this study were transformed into second derivative (2D) spectra by second Savitzky-Golay derivative with window width of 13 points and fitting order of 2. The averaged absorption and 2D spectra were showed in Figure 3. For better comparison with the averaged spectra of populations 7–12, populations of 1–6 were represented with a thin, black and dashed line in panel B and D of Figure 3. From Figure 3A, the spectral intensities for population 2 were higher than the others, however, the variation for six populations were consistent. The baseline shift was removed after 2D pretreatment, and the characteristic peaks of 680, 740, 815, 840, 880, and 920 nm could be seen from Figure 3B. The absorption peak around 680 nm is due to the decrease of chlorophyll content (24). The absorption peak near 740 nm is related to the third-overtone of O-H, and the absorption peak near 840 nm is assigned to an O-H combination (25, 26). The absorption peak near 815 nm is mostly related to the fourth-overtone N-H, and the absorption peak near 880 nm may be caused by the fourth overtone C-H (27). The absorption peak near 920 nm is interpreted as a combination of the third overtone CH-vibration and the third-overtone CH_2_ vibration (28). In addition, the averaged SSC and TA for every population were also marked in Figure 3. But we could not observe pattern clearly, chemometrics methods should be adopted to interpret and develop the model.

### Pretreatment methods

Near infrared spectroscopy contains not only the target component information, but also other irrelevant information and noise. In the process of developing a robust mathematical model, pretreatment for the spectral data has become a common way to remove irrelevant information (29). The pretreatment algorithms of 2D, SNV, MSC, VSN, and NSR were adopted for finding out the suitable pretreatment method. Two wavelengths of 824 and 916 nm were chosen by NSR algorithm without any physical justification, respectively. The number of latent variables (LVs) was determined by the lowest RMSECV value using 5-fold cross validation method. The optimal number of LVs for SSC and TA were 9 and 7, respectively. As shown in Table 3, the best model for SSC (R${}_{\text{p}}^{2}$ = 0.66, RMSEP = 0.66°Brix) with RPD of 1.73 indicated that this model could screen SSC roughly. But this work also encouraged quality assessment of navel orange to forward. The RMSEP reduction of 17.5% for citrus was inferior to 34% reduction for olive DMC (6). Because citrus fruit size matched well with H100F test window, but single olive size was smaller than F750 test window. This result was superior to 10% reduction using SNV pretreatment method for citrus SSC by a portable device (30). However, several pretreatment methods did not achieve good results in TA prediction. The best model (R${}_{\text{p}}^{2}$ = 0.66, RMSEP = 21.82 g/L) with RPD of 0.33 for TA developed by 2D spectra did not have the predictive ability, this result reconsolidated the conclusion in the literatures (4, 31). It had been noted that the interactance optical geometry for field-portable instrumentation was not appropriate for assessment of the acidity of intact low TA fruit of 1%. Therefore, only SSC model was developed and updated in the following section.

**Table 3:** Calibration (P1–4) and prediction (P5) results of PLSR models for SSC and TA with different pretreatment methods.

**Attribute**	**Pretreatment**	**LVs**	**Calibration**				**Prediction**			
			**R${}_{\text{cv}}^{2}$**	**RMSECV**	**Bias**	**RPD**	**R${}_{\text{P}}^{2}$**	**RMSEP**	**Bias**	**RPD**
SSC (°Brix)	2D	9	0.62	0.64	−0.0045	1.48	0.49	0.80	0.2548	1.43
	2D + SNV	9	0.58	0.67	−0.0024	1.42	0.52	0.78	0.3266	1.46
	2D + MSC	9	0.59	0.66	−0.0040	1.44	0.57	0.74	0.3737	1.54
	**2D** **+** **VSN**	**9**	**0.60**	**0.66**	–**0.0013**	**1.44**	**0.66**	**0.66**	**0.1382**	**1.73**
	2D + NSR	9	0.53	0.71	−0.0080	1.34	0.47	0.95	0.4764	1.20
TA (g/L)	**2D**	**7**	**0.83**	**16.16**	–**0.0079**	**0.77**	**0.66**	**21.82**	**0.6255**	**0.33**
	2D + SNV	7	0.79	17.86	−0.0154	0.70	0.51	26.18	5.2522	0.28
	2D + MSC	7	0.80	17.74	−0.0066	0.70	0.50	26.24	6.7637	0.27
	2D + VSN	7	0.79	17.92	−0.0087	0.71	0.53	25.53	7.1741	0.28
	2D + NSR	7	0.80	17.48	0.0129	0.71	0.54	25.29	−2.7920	0.29

*The best calibration and prediction results for each parameter were shown in bold. P represented population*.

### Variable selection

**Figure 4:**
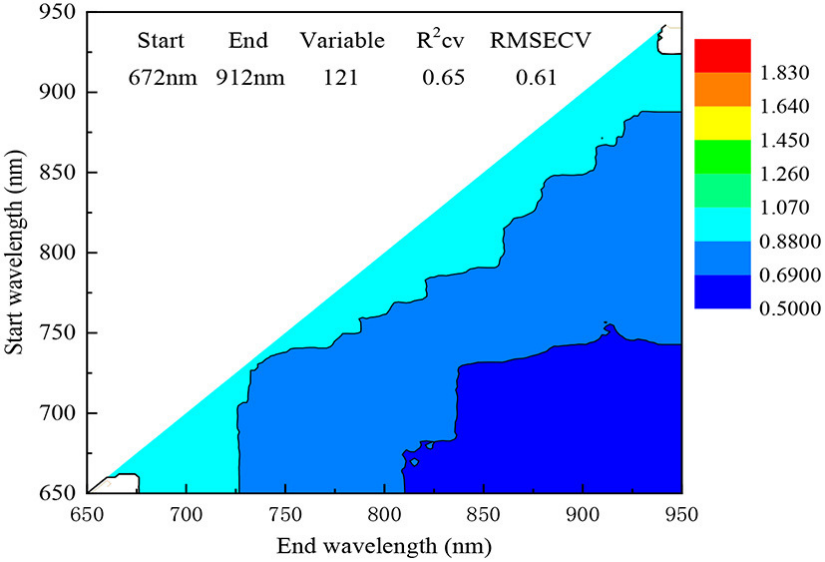
Variation of RMSECV for SSC by variable selection method of window search.

Variable selection is a common method for extracting informative variables or removing irrelevant variables to target component during model development (32). Generally, two strategies involved wavelength combinations and wavelength bands were adopted to build a robust model. The wavelength range for most of the portable fruit selector instruments locates below 1,100 nm due to strong penetration ability and low-cost spectrometer. The variable number often did not exceed three hundred, for example, 151 variables for H100F and 306 for F750, respectively. Therefore, a reasonable window often is recommended, for example 700–930 nm for H100F and 729–975 nm for F750 in predicting SSC of pear and DMC of mango (12, 13). In this work, window search and compared methods of VCPA, IRIV, MC-UVE and SPA were adopted to choose related variables to SSC (Table 4). For SSC, the highest RPD of 1.52 was obtained with the optimal wavelength band of 672–912 nm (121 variables). Figure 4 showed the result of variable selection by window search, and the minimum RMSECV can be obtained in the region of 672–912 nm. However, the best result (R${}_{\text{p}}^{2}$ = 0.55, RMSEP = 0.75°Brix, RPD = 1.52) with variable selection was still inferior to VSN pretreatment with RPD of 1.73. Because VSN attached different weights for adjusting the contribution of every variable to SSC. Further, all methods for variable selection failed based on 2D spectra after VSN pretreated. Therefore, 2D spectra combination of VSN pretreatment in the full region (650–950 nm) were applied for model update.

**Table 4:** Calibration (P1–4) and prediction (P5) results of PLSR models for SSC with different variable selection methods based on 2D or 2D + VSN pretreatment.

**Attribute**	**Pretreatment**	**Method**	**Variable**	**LVs**	**Calibration**				**Prediction**			
					**R${}_{\text{cv}}^{2}$**	**RMSECV**	**Bias**	**RPD**	**R${}_{\text{P}}^{2}$**	**RMSEP**	**Bias**	**RPD**
SSC (°Brix)	2D	None	151	9	0.62	0.64	−0.0045	1.48	0.49	0.80	0.2548	1.43
	2D	VCPA	10	3	0.56	0.69	−0.0054	1.38	0.45	0.83	−0.1752	1.37
	2D	IRIV	30	5	0.62	0.63	−0.0022	1.51	0.54	0.76	0.1715	1.50
	2D	MC-UVE	45	4	0.59	0.66	−0.0018	1.44	0.45	0.84	0.1113	1.36
	2D	SPA	24	11	0.60	0.65	−0.0019	1.46	0.48	0.81	0.1481	1.41
	**2D**	**Window search**	**121**	**9**	**0.65**	**0.61**	–**0.0023**	**1.56**	**0.55**	**0.75**	**0.1016**	**1.52**
	2D	Previous report	116	7	0.62	0.64	−0.0051	1.48	0.51	0.79	0.1534	1.44
	**2D** **+** **VSN**	**None**	**151**	**9**	**0.60**	**0.66**	–**0.0013**	**1.44**	**0.66**	**0.66**	**0.1382**	**1.73**
	2D + VSN	VCPA	11	6	0.55	0.69	−0.0056	1.38	0.55	0.76	−0.0158	1.50
	2D + VSN	IRIV	41	6	0.60	0.65	−0.0019	1.46	0.63	0.68	0.1429	1.68
	2D + VSN	MC-UVE	35	10	0.55	0.69	−0.0038	1.38	0.40	0.87	−0.1349	1.31
	2D + VSN	SPA	27	11	0.59	0.65	−0.0040	1.46	0.56	0.75	−0.0714	1.52
	2D + VSN	Window search	129	9	0.61	0.64	−0.0020	1.48	0.56	0.75	−0.1605	1.52
	2D + VSN	Previous report	116	9	0.52	0.71	−0.0026	1.34	−24.99	5.75	1.4920	0.20

*Previous report recommended wavelength band of 700–930 nm for H100F instrument in predicting SSC of pear by Choi et al. (12). The best result in calibration and prediction for each parameter was bolded. P represented population*.

### Model update

**Figure 5:**
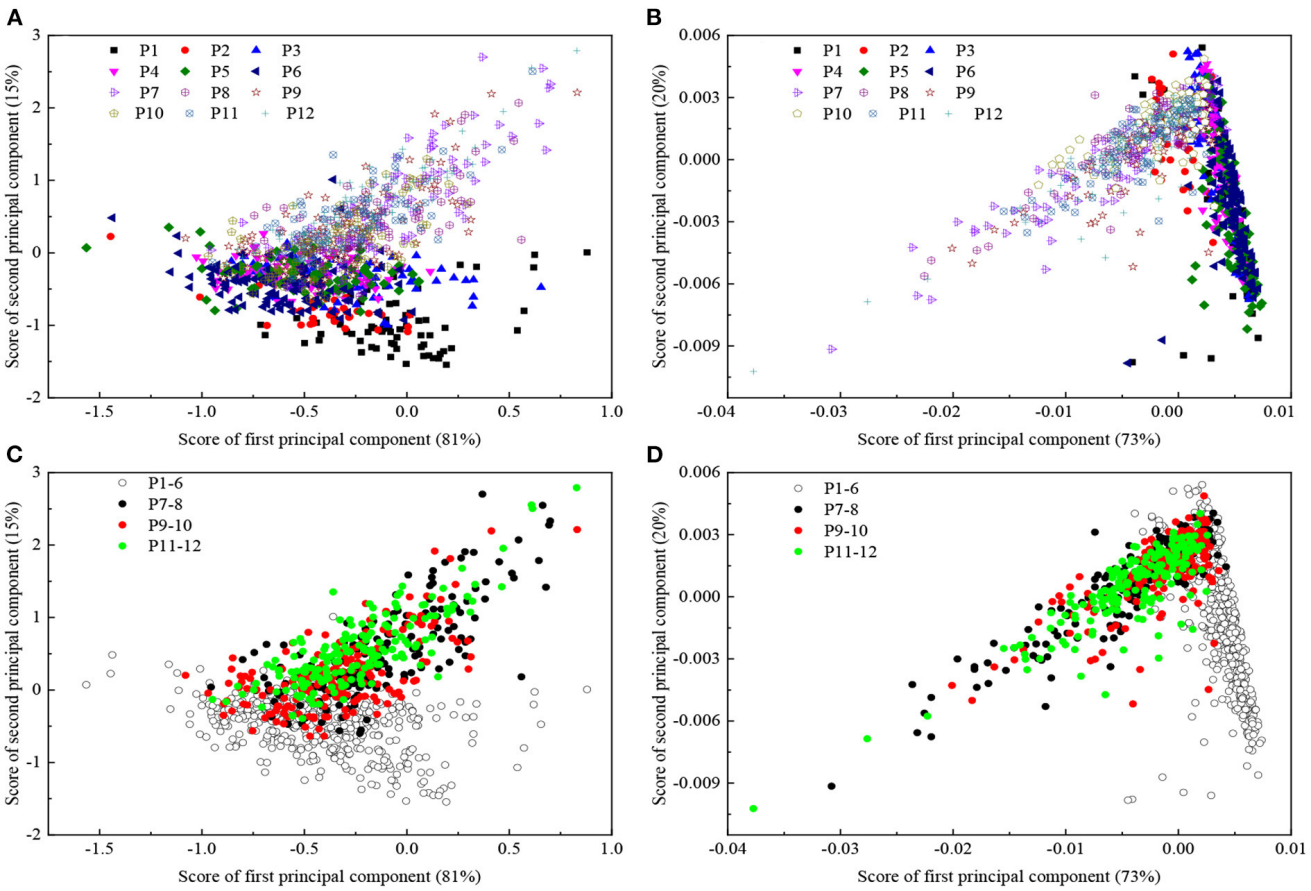
Plots of principal component analysis score for absorption **(A,C)** and 2D spectra **(B,D)**. P1–6, P7–8, P9–10 and P11–12 were illustrated using open white circles with black contour, black closed circles, red closed circles and green closed circles in panel **(C,D)**. P represented population.

The samples in calibration set may not cover with new samples well in prediction set, and the performance of the model will decrease (14). Therefore, model update is a crucial routine for practical application of the portable NIRS instrument. The principle of the model update is to figure out the pattern between calibration and prediction sets. So 2D spectra in the region of 650–950 nm were applied for SSC model update in this section. The score plots of principal component analysis (PCA) for absorption and 2D spectra were shown in Figure 5. The update and prediction sets were designed as shown in Figure 1. The update set of populations 9–10 and prediction set (populations 7–8, 11–12) were used as a negative group during external validation because the region of populations 9–10 could not cover the populations 7–8 and 11–12 well. The methods of DOP, OSC, SBC and MU were employed to update the model, respectively.

DOP method is an extension of EPO (19), which removes the variation in spectra caused by the population change using EPO framework. OSC was compared because of good spectra correction ability was achieved in the literature (21). SBC was also considered because it provided a direct correction routine based on model parameters (18, 33). The number of the samples adding into the calibration set was determined *via* observing the change of the RMSEP (16). For internal and external validation sets, 40 and 30 fruit samples were used as the standard samples in the update sets, which were added into the original calibration set or corrected spectra. K, the parameter of DOP, was optimized from 1 to 20, was listed in Table 5.

**Table 5:** Model update results for H100F portable NIRS instrument.

**Method**	**Parameters**	**Calibration**					**Prediction**				
		**Population**	**R${}_{\text{cv}}^{2}$**	**RMSECV**	**Bias**	**RPD**	**Population**	**R${}_{\text{P}}^{2}$**	**RMSEP**	**Bias**	**RPD**
None	LVs = 9	1–4	0.60	0.66	−0.0013	1.44	5	0.66	0.66	0.1382	1.73
None	LVs = 9	1–4	0.60	0.66	−0.0013	1.44	9–12	−1.92	2.10	1.6978	0.62
None	LVs = 9	1–4	0.60	0.66	−0.0013	1.44	7–8, 10–11	−2.52	2.27	1.7183	0.60
None	LVs = 9	1–4	0.60	0.66	−0.0013	1.44	7–8, 11–12	−3.79	2.58	2.1336	0.52
OSC	LVs = 7	1–4	0.44	0.77	−0.0030	1.23	5	0.69	0.63	0.0369	1.81
OSC	LVs = 7	1–4	0.44	0.77	−0.0030	1.23	9–12	0.08	1.18	0.0839	1.13
OSC	LVs = 7	1–4	0.44	0.77	−0.0030	1.23	7–8, 10–11	0.14	1.12	0.1031	1.22
OSC	LVs = 7	1–4	0.44	0.77	−0.0030	1.23	7–8, 11–12	0.05	1.20	0.4515	1.12
MU	LVs = 9	1–4 + (6)**	0.61	0.66	−0.0036	1.41	5	0.64	0.67	0.3178	1.70
**MU**	**LVs** **=** **9**	**1–4** **+** (7–8)**	**0.55**	**0.74**	**0.0044**	**1.55**	**9–12**	**0.54**	**0.83**	**−0.0632**	**1.60**
MU	LVs = 9	1–4 + (9, 12)**	0.56	0.74	−0.0011	1.34	7–8, 10–11	0.52	0.84	−0.0300	1.63
MU	LVs = 9	1–4 + (9–10)**	0.59	0.73	−0.0016	1.37	7–8, 11–12	0.31	0.98	0.5340	1.37
SBC	LVs = 9	1–4 + (6)^*#^	0.60	0.66	−0.0013	1.41	5	0.71	0.60	0.0577	1.90
SBC	LVs = 9	1–4 + (7–8)^*#^	0.60	0.66	−0.0013	1.55	9–12	0.55	0.84	−0.0129	1.58
**SBC**	**LVs** **=** **9**	**1–4** **+** (9, 12)^*#^	**0.60**	**0.66**	**−0.0013**	**1.34**	**7–8, 10–11**	**0.52**	**0.83**	**0.0152**	**1.65**
**SBC**	**LVs** **=** **9**	**1–4** **+** (9–10)^*#^	**0.60**	**0.66**	**−0.0013**	**1.37**	**7–8, 11–12**	**0.33**	**0.96**	**0.5012**	**1.40**
**DOP**	**LVs** **=** **9, K** **=** **6**	**1–4** **+** (6)*	**0.61**	**0.65**	**−0.0032**	**1.46**	**5**	**0.73**	**0.58**	**0.0261**	**1.97**
DOP	LVs = 9, K = 6	1–4 + (7–8)*	0.54	0.70	−0.0035	1.64	9–12	0.49	0.88	0.0114	1.51
DOP	LVs = 9, K = 6	1–4 + (9, 12)*	0.57	0.68	−0.0031	1.46	7–8, 10–11	0.47	0.88	0.0636	1.56
DOP	LVs = 9, K = 5	1–4 + (9–10)*	0.56	0.68	−0.0032	1.47	7–8, 11–12	0.20	1.05	0.6199	1.28

The PLSR model for SSC was developed based on 2D + VSN pretreatment. The best result in calibration set and prediction set for SSC was bolded. RPD value of MU was different with None, OSC, SBC and DOP. Because the number in predicting set was different. The symbol (^*^) represents the update set for spectra correction between the original calibration set and the update set using DOP method. The symbol (^*#^) represents the update set for model parameters correction between the original calibration set and the update set using SBC method. The symbol (^**^) represents the update set adding to the original calibration set for recalibration.

**Figure 6:**
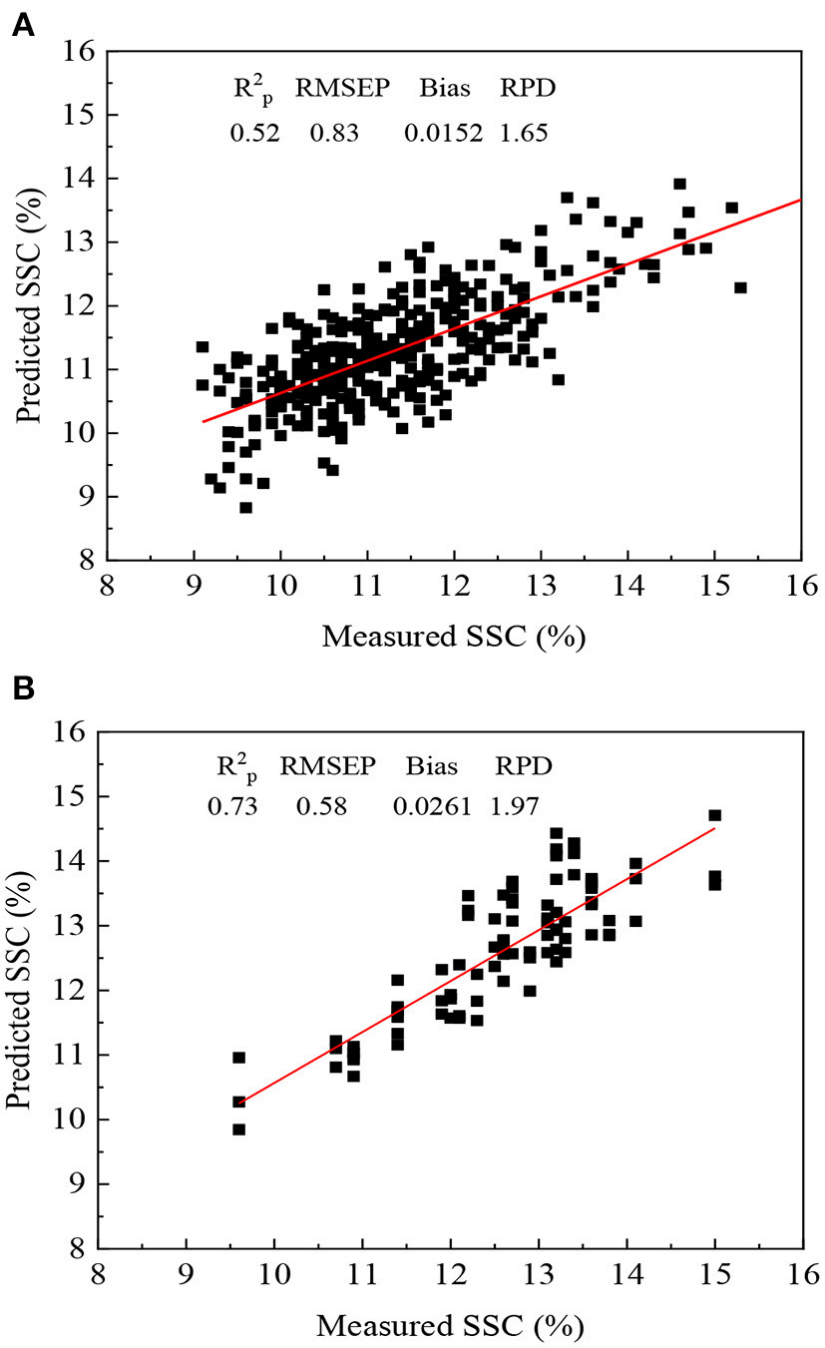
Prediction results of the PLSR model of SSC after SBC **(A)** and DOP **(B)** in predicting populations 7–8 and 10–11 (*n* = 360) and populations 5 (*n* = 87), respectively.

For the internal validation (population 5), the best result was obtained using DOP method followed by SBC, OSC and MU with R${}_{\text{p}}^{2}$ of 0.73, RMSEP of 0.58 °Brix and RPD of 1.97 (Table 5; Figure 6B). It could be found from rows 2 to 4 in Table 5 that direct application of the PLSR model failed completely in external validation (RPD < 1). It should be noted that the PLSR model developed with Newhall navel orange only allowed to work on the new batch (Lunwan navel orange) after updating. For the three external validation sets, methods of MU, SBC and DOP for the two positive groups (populations 9–12 and 7–8, 10–11) achieved better results than the negative group (populations 7–8 and 11–12) except OSC. The negative group yielded always the worst results, because the performance for model update was determined by the representation of the update set to the prediction set (34). The prediction set could not be covered well by the update set in Figures 5C,D. The best results in predicting populations 9–12, populations 7–8 and 10–11 and populations 7–8 and 11–12 were achieved by MU and SBC, respectively (Table 5; Figure 6A).

The internal validation (population 5) and two positive validation (population 9–12, 7–8, and 10–11) sets with R${}_{\text{p}}^{2}$ of 0.52–0.73 and RMSEP of 0.58–0.83 were superior to the previous report in predicting citrus SSC for R_p_ of 0.4 and RMSEP of 1.16 Brix (30). However, our result was inferior to the reports in the literatures (35–37) (Table 6). Because an external prediction set with different variety and region was still a challenge for the portable NIRS instrument practical application. Methods of SBC and MU were also recommended for easy operation for the use.

**Table 6:** The previous reports on assessment of citrus quality using NIRS instrument.

**Objective**	**Attribute**	**Method**	**Result**	**References**
Citrus	SSC	SNV-SPA	Rp = 0.92, RMSEP = 0.57	(30)
Orange	SSC	SpectraNet−53	Rp = 0.40, RMSEP = 1.16	(35)
Mandarin	Sugar degree	BPNN	Rp = 0.87, RMSEP = 0.74	(36)
Citrus	SSC	VABPLS	Rp = 0.82, RMSEP = 0.60	(37)

*Rp is correlation coefficient of prediction; VABPLS is variable adaptive boosting partial least squares; SpectraNet−53 is a deep residual learning architecture; BPNN is backward propagation neural network*.

## Conclusions

Model development of pretreatment and variable selection methods and model upgrade methods of MU, SBC, DOP, and OSC were investigated to ensure the predictive ability of portable NIRS instrument for SSC and TA of citrus fruit. The best models could predict SSC but not TA according to the critical limit of RPD of one. For model development, the combinations of VSN and second derivative pretreatment in the full region (650–950 nm) achieve the best results with R${}_{\text{p}}^{2}$ of 0.66, RMSEP of 0.66 °Brix and RPD of 1.73 in predicting SSC. For model update, MU and SBC achieved the best results in predicting SSC for two external validation sets with R${}_{\text{p}}^{2}$, RMSEP and RPD of 0.54, 0.83 °Brix, 1.60 and 0.52, 0.83 °Brix, 1.65, respectively. In addition, this work provided a case for model development and upgrade for the portable NIRS instrument in practical application of fruit quality assessment.

## Data availability statement

The original contributions presented in the study are included in the article/Supplementary material, further inquiries can be directed to the corresponding author.

## Author contributions

XS: conceptualization, methodology, review and editing, project administration, funding acquisition, supervision, and resources. DD: spectra collection, formal analysis, and original draft. JL: sample collection and analysis. SF: instrument support. All authors contributed to the article and approved the submitted version.

## Funding

This work was supported by Jiangxi Province Technology Innovation Guidance Project (No. 20212BDH81008).

## Conflict of interest

Author SF is employed by Beijing Sunlight Yishida Technology Co., Ltd. The remaining authors declared that the research was conducted in the absence of any commercial or financial relationships that could be construed as a potential conflict of interest.

## Publisher's note

All claims expressed in this article are solely those of the authors and do not necessarily represent those of their affiliated organizations, or those of the publisher, the editors and the reviewers. Any product that may be evaluated in this article, or claim that may be made by its manufacturer, is not guaranteed or endorsed by the publisher.
