# Supplementary material for: Model development and update of portable NIRS instrument for assessment of internal quality attributes of two navel orange varieties
Source: Front Nutr. 2022 Aug 24;9:976178. doi: 10.3389/fnut.2022.976178 (PMC9450129; doi:10.3389/fnut.2022.976178)
Supplement: Supplementary file 1 [file Table_1.DOCX]

**Declaration of Competing Interest**

The authors declare that they have no known competing financial interests or personal relationships that could have appeared to influence the work reported in this paper.
